# Understanding the Connection Between Common Stroke Comorbidities, Their Associated Inflammation, and the Course of the Cerebral Ischemia/Reperfusion Cascade

**DOI:** 10.3389/fimmu.2021.782569

**Published:** 2021-11-15

**Authors:** Łukasz Przykaza

**Affiliations:** Laboratory of Experimental and Clinical Neurosurgery, Mossakowski Medical Research Institute, Polish Academy of Sciences, Warsaw, Poland

**Keywords:** adhesion molecules, cerebral ischemia/reperfusion, inflammation, neurovascular unit, stroke comorbidities

## Abstract

Despite the enormous progress in the understanding of the course of the ischemic stroke over the last few decades, a therapy that effectively protects neurovascular units (NVUs) and significantly improves neurological functions in stroke patients has still not been achieved. The reasons for this state are unclear, but it is obvious that the cerebral ischemia and reperfusion cascade is a highly complex phenomenon, which includes the intense neuroinflammatory processes, and comorbid stroke risk factors strongly worsen stroke outcomes and likely make a substantial contribution to the pathophysiology of the ischemia/reperfusion, enhancing difficulties in searching of successful treatment. Common concomitant stroke risk factors (arterial hypertension, diabetes mellitus and hyperlipidemia) strongly drive inflammatory processes during cerebral ischemia/reperfusion; because these factors are often present for a long time before a stroke, causing low-grade background inflammation in the brain, and already initially disrupting the proper functions of NVUs. Broad consideration of this situation in basic research may prove to be crucial for the success of future clinical trials of neuroprotection, vasculoprotection and immunomodulation in stroke. This review focuses on the mechanism by which coexisting common risk factors for stroke intertwine in cerebral ischemic/reperfusion cascade and the dysfunction and disintegration of NVUs through inflammatory processes, principally activation of pattern recognition receptors, alterations in the expression of adhesion molecules and the subsequent pathophysiological consequences.

## 1 Introduction

Ischemic stroke is a serious clinical and socioeconomic problem, especially among the aging populations of industrialized countries. Despite the enormous progress in the understanding of the course of the ischemic cascade over the last few decades, a neuroprotective therapy that effectively protects neurovascular units (NVUs) and significantly improves neurological functions in stroke patients has still not been achieved. The reasons for this state are still far from fully clear. Admittedly, the ischemia and reperfusion cascade is a highly complex process that consists of many elements of a diverse biological nature, and comorbid stroke risk factors strongly worsen stroke outcomes and likely make a substantial contribution to the pathophysiology of the ischemic/reperfusion cascade, enhancing difficulties in treatment (1, 2). The most common risk factors found in stroke patients are arterial hypertension, diabetes mellitus and hyperlipidemia. All of these factors cause low-grade inflammation and microcirculatory disturbances in many organs, and also – in the brain. Therefore, in this paper, a strong emphasis was placed on the perspective that the main link unifying the ischemia/reperfusion cascade and these risk factors are inflammatory processes that already initially disrupt the proper functions of NVUs, before a stroke onset, and participate in the course of a stroke pathomechanism.

For many years, evidence has increased for an ambiguous, destructive, protective and repairing role of some inflammatory cells in the course of a stroke, although many early studies in this field unequivocally showed that the infiltration of the ischemic brain by inflammatory cells enlarged the extent of post-ischemic damage through subsequent edema or intracranial hemorrhage. This ambiguity does not undermine the possibility of therapeutic targeting of the ischemic inflammatory process; on the contrary, in the future, appropriate immunomodulation may halt damaging processes and/or enhance the protective or repair functions of some of their effectors. However, despite ambiguous details, the negative role of inflammatory processes in focal and global cerebral ischemia/reperfusion, intracranial hemorrhages, or mechanical brain injuries is brought to the fore. Clinically, in the course of ischemic stroke, a higher level of inflammation is a significant prognostic factor for worse treatment outcomes (3). In contrast, animal experimental studies on neuroprotection still do not consider the coexisting risk factors for stroke in humans as an experimental standard. This is probably an important cause of the failure of attempts to introduce neuroprotection into clinical practice. Accompanying risk factors already presensitize neurovascular units to the ischemic/reperfusion cascade. When the cascade occurs, it encounters NVUs with their already initially impaired functionality and consistently worsens cerebral perfusion at the level of the microcirculation in the ischemic penumbra. Considering this situation in basic research may prove to be crucial for the success of future clinical trials of neuroprotection and immunomodulation.

This review of the literature focuses on the mechanism by which coexisting common risk factors for stroke intertwine in ischemic/reperfusion and the dysfunction and disintegration of NVUs through inflammatory processes, principally activation of pattern recognition receptors, alterations in the expression of adhesion molecules and the subsequent pathophysiological consequences.

For the review, the PubMed electronic database was searched using the following keywords: adhesion molecules, inflammation, ischemic stroke, pattern recognition receptors, penumbra, reperfusion, stroke risk factors. Experimental, clinical and review publications from 1971 to 2021 were cited, with a predominance of those published after the year 2000.

## 2 The Cerebral Ischemia/Reperfusion Cascade and Stroke Comorbidities

Arterial hypertension, diabetes mellitus, and hyperlipidemia are well-known modifiable risk factors for the development of ischemic stroke in humans. Currently, an epidemic increase in the incidence of these diseases is observed in the Western world. In many countries, approximately 50% of the population over 60 years of age has been diagnosed with hypertension, and this prevalence is actually increasing (4). Elevated diastolic or systolic blood pressure, or both parameters, is a major risk factor for stroke development (5, 6). Similarly, diabetes mellitus shows a worrying trend of increasing morbidity, despite the presence of many pro-health social campaigns. Type 2 diabetes, until recently considered mainly as a disease of elderly age, is now responsible for approximately 4-5% of premature deaths (7). Approximately 50% of people with diabetes signs delayed end-organ/system damages and as many as 40% of subjects have vascular complications, both result from the silent progression of the disease before its diagnosis (8). Hyperlipidemia (elevated blood lipid/lipoprotein levels) also severely affects various age groups in wealthy Western societies. As an example in the United States of America, ~33.5% of adults aged ≥20 years have elevated low-density lipoprotein cholesterol levels above 160 mg/dL (9). In adults, hyperlipidemia is an important risk factor for the development of cardiovascular diseases and stroke (10, 11).

Molecular mechanisms involved in inflammation, e.g., in general, an activation of the pro-inflammatory genes and an increase in the expression of adhesion molecules, in addition to their well-documented role in the ischemic cascade in the brain, can now also be reasonably considered an important connecting bridge between the above risk factors for ischemic stroke and the unfavorable prognosis in the event of its occurrence. Hypertension and diabetic hyperglycemia can worsen the prognosis of stroke through endothelial dysfunction and increased leukocyte adhesion to blood vessels and other pathologies affecting the functions of NVUs. Moreover, chronically elevated blood cholesterol levels also result in endothelial disturbances and increased adhesion of leukocytes and platelets during ischemia/reperfusion (12–14). These observations constitute important clinical indications for research in the field of ischemic stroke neuroprotection, vasculoprotection and immunomodulation, and for widespread preclinical studies with animal models with modeled risk factors for stroke in humans.

Based on the current knowledge, to understand the mechanistic links how the common risk factors for stroke may worsen its course through amplification of the disintegration and dysfunction of NVUs, we first need to review the mechanisms of ischemic/reperfusion cascade and ischemic inflammation in light of this regard.

### 2.1 The Mechanisms of NVUs Damage in the Course of the Focal Cerebral Ischemia/Reperfusion Cascade

During an ischemic stroke, a decrease in the patency of the plugged artery lumen leads to a local decrease in the volume of blood flow in the brain area supplied by it. This decline is not uniform across the entire ischemic region. On the basis of the degree of blood flow reduction, it is possible to distinguish the infarct core (< 20% of basal blood flow) and the penumbra, where the collateral circulation maintains the blood flow at the level of ~40% of the basal blood flow (15). During ischemic oxygen-glucose deprivation in the cells located in the infarct core, there is a strong decrease in the rate of oxidative phosphorylation, a decrease in intracellular ATP (adenosine 5*′-*triphosphate) concentration, and consequently a decrease in the activity of Na^+^/K^+^-ATPase and a disturbance in the ATP-dependent transport of K^+^ and Na^+^ ions. The ion homeostasis of the cells in the infarct core is lost. The outflow of K^+^ from cells and the inflow of Na^+^ into the cytosol causes cellular depolarization. High cytosolic concentrations of Na^+^ and Cl^-^ result in the development of strong cytotoxic edema (16, 17). Depolarized neurons in the infarct core are not able to restore their resting potentials, and they die within a few minutes as a result of energetic collapse, a loss of ion homeostasis, cytotoxic edema, proteolysis, cytoskeleton disintegration, lipolysis and cell membrane fragmentation (18). The loss of energy/ion homeostasis disrupts the communication of neurons with glial and endothelial cells. Astrocytes exposed to glutamate toxicity, similar to neurons, suffer from cytotoxic edema, calcium overload and mitochondrial depolarization, followed by free radical damage (19, 20).

Currently, it is commonly accepted that damage to NVUs in the penumbra occurs as a consequence of a combination of numerous factors of a diverse nature – such as excitotoxicity, peri-infarct depolarizations (PIDs), free radical stress, apoptosis, inflammatory processes, cerebral blood vessel damage, and microcirculatory disturbances during ischemia and reperfusion. It seems, however, that glutamate excitotoxicity and peri-infarct depolarizations are the most primary (21, 22). Within 6–8 hours, the penumbra is recruited to the infarct core (23). According to the excitotoxicity hypothesis, a high concentration of glutamate diffuses from the infarct core to its edge and hyperactivates postsynaptic N-methyl-D-aspartate receptors (NMDA-Rs) in penumbra neurons (24, 25). In response to excessive, long-term stimulation of NMDA-Rs, there is an excessive intracellular influx of Na^+^ and Ca^2+^ ions. Highly elevated intracellular concentrations of Ca^2+^ initiate a number of pathways, leading to the death of neurons (26). At the same time, astrocytes internalize glutamate transporter-1 (GLT-1) – transporters most importantly involved in the reuptake of glutamate from the synaptic cleft, which leads to a high concentration of glutamate in the interstitial fluid. The downregulation of GLT-1 enhances and prolongs the excitation of postsynaptic neurons (27, 28). Moreover, a sharp increase in the activity of astrocytic cAMP/PKA (3′,5′*-*cyclic adenosine monophosphate/protein kinase A) signaling leads to the phosphorylation of aquaporin-4 (AQP4), which ultimately increases the permeability of the cell membrane to water and promotes cytotoxic edema of astrocytes and mechanical pressure on microvessels (29). These astrocytic metabolic disorders (with calcium overload) are the initiation of the astrogliosis. In turn, PIDs are initiated mainly by a high concentration of K^+^ ions diffusing in the extracellular space. These ions come from depolarized/dead neurons of the infarct core and reach high concentrations there as a result of astrocyte dysfunction. PIDs, due to insufficient blood supply and the decoupling of the relationship between the metabolism of neurons and the reactivity of microcirculation in the penumbra, lead to episodes of hypoxia and energetic depletion of neurons in this zone and a loss of their ion homeostasis, which may also cause anoxic depolarizations. The number of PIDs positively correlates with the size of ischemic damage (30). With regard to astrocytic upregulation AQP4, it was shown that this protein significantly contributed to the propagation of depolarization waves by increasing the extracellular concentration of K^+^ (31). Additionally, the downregulation of GLT-1 protein is involved in the propagation of depolarizing waves as well (32). PIDs also damage NVUs as a result of the activation of metalloproteinases (e.g., metalloproteinase-9, MMP-9) and the subsequent weakening of the blood–brain barrier (BBB). The severity of ischemic BBB damage and the degree of vasogenic edema are positively correlated with the number of PID episodes (33).

When a reopening of an arterial vessel occurs, reperfusion takes place, restoring blood flow through the ischemic area. Depending on the duration of ischemia and/or the patient’s health history, reperfusion may bring clinically beneficial as well as unfavorable effects (34). However, reperfusion carries risks, especially after a long episode of ischemia. Some experimental studies have shown that focal ischemia with subsequent reperfusion can cause more extensive brain damage than focal ischemia without reperfusion (35, 36). During reperfusion, disturbances in the regulation of blood flow in the penumbra and oligemic areas are revealed, and reactive oxygen species are produced by the NADPH oxidase (NOX) system, the xanthine oxidase (XO) system, and the mitochondrial enzymatic systems damaged by ischemia (37). The significant presence of the NOX and XO systems has been confirmed in blood vessels (endothelial cells). Hypoxic/reoxygenated endothelial cells show an increase in NOX expression/activity, reactive oxygen species (ROS) production, and adhesion molecule expression, causing vascular injuries. All of these responses can be prevented by treating cells with inhibitors of NOX-activating signaling pathways (38–40). Likewise, for XO, various cytokines released during reperfusion, including IL-1 (interleukin 1), IL-6 (interleukin 6), TNF-α (tumor necrosis factor alpha), and IFN-γ (interferon gamma), increase the expression/activity of the enzyme in various cell types, and inhibitors of XO prevents against vascular injuries (37, 41).

During the reperfusion phase, inflammatory processes evidently begin; activated peripheral leukocytes migrate to brain tissues, contributing to brain tissue damage, the aggravation of BBB disruption and vasogenic edema, as well as hemorrhagic transformation (HT); and blood cellular elements create intravascular conglomerates that impair microcirculation (42, 43).

### 2.2 Inflammatory Processes in the Course of Ischemic Stroke

#### 2.2.1 General View

The dead and dying cells of the brain ischemic area induce inflammatory processes by releasing into their surroundings molecules that activate microglial cells, endothelial cells of the brain blood vessels, astrocytes and, in subsequent stages, infiltrating leukocytes. These molecules are collectively known as DAMPs (danger-associated molecular patterns) and include, e.g., ATP, UTP (uridine 5’-triphosphate), nicotinamide adenine dinucleotide, peroxiredoxins, HMGB1 (high mobility group box 1) and HSP60 (heat shock protein 60) (44). Microglial cells, leukocytes, astrocytes and neurons, and endothelial cells present receptors in their cell membranes that can be stimulated by these molecules. The activation of these receptors triggers a series of processes leading to the expression of pro-inflammatory genes and changes the resting phenotype of microglia into the amoeboid M1. Microglia transform, proliferate, rearrange the cytoskeleton, and acquire the ability to migrate and phagocytose (45). ATP and UTP stimulate P2X7 (P2X purinoceptor 7) purinergic receptors. HMGB1 stimulates CD36 (cluster of differentiation 36), TLR4 (Toll-like receptor 4) and TLR2 (Toll-like receptor 2), RAGE (receptor for advanced glycation end products), and receptors for HSP60. Peroxiredoxins stimulate TLR2 and TLR4 receptors (46–49). As a result of the activation of the aforementioned receptors, the transcription factor NF-κB (nuclear factor kappa B) triggers the expression of genes encoding pro-inflammatory molecules, including IL-1β (interleukin*-*1β), IL-6, IL-8 (interleukin 8), IL-18 (interleukin 18), and TNF-α. Another activated transcription factor involved in the expression of pro-inflammatory genes is AP-1 (activator protein 1), an important activator of IL-1 and TNF-α expression and the promotion of the expression of some adhesion molecules (50–52). The path from TLR4 receptors to NF-κB and AP-1 activation is mainly through the MyD88 protein (myeloid differentiation primary response protein) (53, 54). Apart from cytokines, chemokines such as: MCP-1 (CCL2; monocyte chemoattractant protein*-*1), MIP-1a (CCL3; macrophage inflammatory-1 alpha), CXCL2 (MIP-2, macrophage inflammatory protein 2), RANTES (CCL5; regulated on activation, normal T-cell expressed and secreted), CINC-1 (cytokine-induced neutrophil chemoattractant 1), fractalkine (CX3CL1; C-X3-C motif chemokine ligand 1), and CXCL10 (IP-10; C-X-C motif chemokine ligand 10) are expressed in the ischemic area (55–57). The activation of microglial cells occurs in the first minutes of a stroke. Microglial cells can damage neurons, oligodendrocytes, astrocytes and blood vessels in the early phase, releasing, e.g., ROS. However, in later stages, they may play a protective or repair role (M2 population) due to the release of TGF-beta1 (transforming growth factor beta 1), GDNF (glial cell derived neurotrophic factor) and the anti-inflammatory IL-10 (interleukin 10) (58–60).

Pro-inflammatory processes make the ischemic environment conducive to the migration of immune cells from peripheral blood over time. The most abundant leukocytes to first make the passage from the blood to the brain parenchyma are neutrophils. Experimental studies have shown that neutrophils contribute to the enlargement of the area of damage after infiltration by releasing ROS and metalloproteinases, and by activating own iNOS (inducible nitric oxide synthase) in response to the presence of specific cytokines (61–63). Secondly, circulating monocytes attracted by specific chemokines, penetrate the brain damage through the weakness of BBB. Peripheral monocytes, considering their role in post-ischemic inflammatory processes, can be divided into pro-inflammatory, with the phenotype Ly-6C^high^/CCR2^+^, releasing IL-1β and TNF-α, and anti-inflammatory Ly-6C^low^/CCR2^-^, releasing IL-10 (64, 65). Lymphocytes, another class of leukocytes playing a complex role in the pathogenesis of ischemic damage, appear in the ischemic area 24 hours after the start of reperfusion and reach a peak after seven days. There are several types of lymphocytes involved, of which, for example, CD8^+^ T cells play a negative role, and γδ T cells play a negative role in the late but not the early phase of stroke, substantially through the production of pro-inflammatory IL-17 (interleukin 17) and thus supporting the influx of neutrophils (66, 67).

CD8^+^ T lymphocytes and Natural Killer cells (NK) enhance the infiltration of the brain parenchyma by recognizing the IL-15 (interleukin 15) signal produced by activated astrocytes. The increase in IL-15 expression during ischemia significantly contributes to inflammatory processes and enlarges the area of post-ischemic brain damage (68). The activation of astrocytes is important and one of the first processes during ischemic inflammation. During this activation astrocytes undergo morphological changes, principally hyperplasia and hypertrophy, and one of the molecular markers of this process is increased expression of GFAP (glial fibrillary acidic protein) (69). Moreover, changes in AQP4 expression are observed, whose directivity depends on the timing of the ischemic cascade and on the ischemic region location. There is a strong increase in the expression of this protein at 1 hour of ischemia in the infarct core and at 1 and 48 hour of ischemia in the penumbra, which correlates with cytotoxic edema (70). In the core area of the striatum 24 hours after ischemia/reperfusion, the perivascular AQP-4 level was shown to be significantly decreased and had no recovery tendency. In the ischemic core of the cortex, however, the presence of AQP-4 is obviously reduced at 24 h and gradually recovers at 72 h after reperfusion (71). Overall, an increase in AQP4 expression is correlated with the cytotoxic edema of astrocytes, and a decrease in AQP4 expression is correlated with vasogenic cerebral edema (72).

From the above data, a very complex landscape of the inflammation involved in the pathophysiology of ischemic stroke emerges. In general, a clinically useful marker of the advancement of the ongoing inflammatory processes is CRP (C-reactive protein), and in stroke patients the concentration of this marker is significantly elevated. On the one hand, this increase in CRP indicates the presence of a serious disease, and on the other hand, it is an important prognostic marker of the outcomes of stroke treatment, probably also because CRP can directly cause changes in the endothelium – such as increasing of the expression of adhesion molecules, mediating the invasion of peripheral leukocytes into the brain tissues (73–75).

Inflammatory processes are a double-edged sword and play both a positive and negative role in the development of ischemic cerebral damage, depending on their phase, the type of actually involved inflammatory cells, and the patient’s health history. This presents a challenge for research into the mechanisms and future therapy of ischemic stroke. However, there are valid immunological goals for research on neuroprotection and vasculoprotection. The penetration of leukocytes from blood into the damaged brain involves several stages, mainly determined by: 1) the appearance of pro-inflammatory cytokines and chemokines in damaged tissues, and 2) the expression of adhesion molecules in microvascular endothelial cells and their presentation on the surface of the cell membranes.

The negative effects of leukocyte invasion into the ischemic brain may cause secondary damage within the penumbra. Several mechanisms of secondary brain injury have been proposed. Three of them that appear to be the most important are a secondary reduction in cerebral blood flow during reperfusion, the permeabilization of blood vessels, and the production of oxygen (ROS) and nitrogen (RNS – reactive nitrogen species) free radicals causing general tissue damage. Secondary reduction of blood flow in the vessels of the microcirculation (no-reflow effect) may be, on the one hand, an effect of cytotoxic swelling of astrocytes and the compression of their foot-ends on arterioles, and, on the other hand, a result of leukocyte adhesion to the inner surfaces of blood vessel walls, in particular the endothelial cells of venules. The adherence of leukocytes and platelets to each other causes the formation of a specific network inside the lumen of the vessel, which is additionally strengthened by fibrin, which immobilizes erythrocytes. In this way, conglomerates/aggregates of blood cells, or platelet-leukocyte aggregates (PLAs), are formed, and their adherence to the inner walls of the microvessels narrows the vascular lumen, disrupting blood microcirculation as a result. PLAs may also detach from the site of their formation and enter the general bloodstream (76). Therefore, it seems that when the therapy achieves only the elimination of the occlusion of a larger, blood-supplying vessel, which is the cause of ischemia, incomplete restoration of penumbral microcirculation may significantly weaken the treatment.

Activated inflammatory cells, mainly neutrophils and macrophages, release large amounts of metalloproteinases, in particular, gelatinases: MMP-9, MMP-2 (metalloproteinase-2), which is also released by activated astrocytes, and collagenase: MMP-13 (metalloproteinase-13). These enzymes have neurotoxic properties, and they degrade the integrity of the BBB, among other ways, by digesting the basal membrane of the vessels. This can lead to HT during reperfusion, contribute to the enlargement of the developing vasogenic cerebral edema, and further increase the influx of leukocytes into the brain. These processes significantly reduce the clinical capabilities of using tissue plasminogen activators and the mechanical recanalization of the plugged artery (77, 78).

Free radicals, including ROS, are produced in high concentrations by leukocytes to fight microorganisms. Although the post-ischemic inflammatory process develops under sterile conditions, stimulated leukocytes produce ROS, similar to their native response to pathogens. One of the most reactive free radicals is the superoxide anion radical $\left({\text{O}}_{2}^{-}\right)$ . The major ${\text{O}}_{2}^{-}$ producing enzyme system found in inflammatory cells such as neutrophils, lymphocytes and macrophages is NOX (79, 80). As mentioned, NOX is also widely abundant in the endothelial cells of blood vessels and may be stimulated by cytokines. Superoxide anion radicals and NO (nitric oxide) show strong mutual chemical affinity. During ischemia/reperfusion, iNOS is stimulated because of the presence of inflammatory IL-1β and TNF-α molecules in ischemic tissues – which increases the expression of the iNOS isoform in endothelium and infiltrating neutrophils, producing high concentrations of NO (62, 81). Other types of NO synthases are additionally stimulated during a stroke, e.g., endothelial isoform (eNOS) is stimulated by an increase in the intracellular concentration of Ca^2+^ ions, which is considered a protective phenomenon as it may improve the blood flow in the penumbra (82). NO and ${\text{O}}_{2}^{-}$ react with each other to form peroxynitrite (ONOO^-^), a very chemically reactive free radical with a long half-life. Similar to other free radicals, peroxynitrite damages, e.g., nucleic acids, proteins and lipids. It has been observed that microvessels located in the ischemia/reperfusion region have numerous zones containing products of reaction with ONOO^-^, such as 3-nitrotyrosine, a marker of protein nitrosative damage (this type of damage is also observed in other brain tissues affected by ischemia/reperfusion). Moreover, the formation of ONOO^-^ reduces the bioavailability of NO, which is the reason for the disturbance in the NO-dependent mechanism of regulation of the tension of the vascular wall and is a marker of endothelial dysfunction (83, 84).

#### 2.2.2 Adhesion Molecules – Their Role in Ischemic Neuroinflammation

In response to ischemia/reperfusion, activated endothelial cells begin to express adhesion molecules and, within a few hours, display them in large numbers on their cellular membrane; as a result, they start to recruit large numbers of myeloid cells to their surface, mainly neutrophils as well as, in a smaller number, macrophages, which then pass into post-ischemic tissues during reperfusion. The endothelium can be activated by IL-1β and TNF-α molecules (85, 86). Leukocyte capture and rolling on the endothelial surface, firm adhesion and transmigration through the blood vessel wall (diapedesis) constitute three stages of the process of entering the tissues, which is determined by the expression of adhesion molecules on the membranes of endothelial cells and white blood cells.

Among the adhesion molecules, three classes can be distinguished: the selectins, which include, e.g., P-selectin, E-selectin and L-selectin; the integrins, which include, e.g., CD18/CD11a (LFA-1, lymphocyte function associated antigen 1), CD18/CD11b (Mac-1, macrophage-1 antigen), CD18/CD11c (integrin alpha X beta 2), α4ß1 (VLA-4, very late antigen-4) and integrin α6ß4; and the immunoglobulin superfamily, which include, e.g., ICAM-1 (intercellular adhesion molecule 1), ICAM-2 (intercellular adhesion molecule 2), VCAM-1 (vascular cell adhesion molecule 1), PECAM-1 (platelet endothelial cell adhesion molecule-1), and MAdCAM-1 (mucosal vascular addressin cell adhesion molecule 1) (87).

Selectins mediate non-tight interactions (mainly rolling) between the endothelium and leukocytes. P-selectin is expressed in endothelium and platelets. In endothelial cells, P-selectin is deposited in Weibel-Palade bodies, and in platelets, it forms cytoplasmic alpha granules. E-selectin is expressed only in the endothelium, while L-selectin is expressed in the endothelium and leukocytes. Leukocytes express the SLeX oligosaccharide antigen (Lewis-X sialyl), which binds to P- and E-selectin, conditioning the rolling stage (88). Experimental work on models of focal cerebral ischemia has shown that blocking P-selectin or E-selectin with monoclonal antibodies or the knockout of the genes of these molecules reduced post-ischemic brain damage and improved the motor skills of tested animals. P-selectin blockade with specific monoclonal antibodies also reduced the number of no-reflow phenomena during reperfusion. In addition, the overexpression of P-selectin or E-selectin magnified post-stroke injury (89, 90). However, in the global cerebral ischemia model, the blockage of P-selectin with specific monoclonal antibodies increased the mortality of the animals used in the experiment (91). These inconsistencies perhaps result from differences between experimental stroke models. Leukocytes have a specific antigen for P-selectin – PSGL-1 (P-selectin glycoprotein ligand-1) deployed to their cell membranes. This interaction likely participates in the formation of PLA aggregates and the secondary activation of both types of cells (92). The use of sCRsLex, an inhibitor of platelet-leukocyte adhesion interactions, reduced the volume of post-stroke cerebral infarction in experimental animals (93). Very promising observations of a reduction in post-stroke damage have been made by administering E-selectin intranasally to spontaneously hypertensive stroke-prone rats prior to focal cerebral stroke with reperfusion. In addition, a decrease in the incidence of spontaneous strokes has been demonstrated in this strain of rats after the intranasal administration of E-selectin (94, 95). Thus, there is expectation for the development of a specific vaccine that could, for example, improve prognosis in patients at risk. However, in some studies of patients with ischemic stroke to date, inconsistent results in measurements of serum E-selectin levels have been obtained. In the study of patients with stroke symptoms, an increase in the concentration of E-selectin was observed for 24 hours, while in other studies, no such phenomenon was observed (96). Inconsistencies also appear in experimental research. Experiments in rats with spontaneous hypertension have shown that functional blocking of the E-selectin molecule reduced post-stroke damage in a model of transient but not permanent focal cerebral ischemia (97). The role of L-selectin in stroke is unclear. L-selectin has been shown to mediate the process of neutrophil rolling, but the blockade of L-selectin with monoclonal antibodies did not result in neuroprotection in a focal stroke model in rabbits (98, 99).

Another class of adhesion molecules are integrins, which are heterodimeric cell membrane glycoproteins consisting of two subunits: α and β, bounded by weak interactions. There are several β subunit subtypes, e.g., β1, β2, β3, and β4, which represent a specific subclass of molecules within which the α subunit changes (100). Examples of integrins containing β1 and β4 subunits are α1β1, expressed in microvascular endothelia and astrocytes, and α6β4, found at the perivascular end*-*feet of astrocytes. These integrins play a very important role in the brain, taking part in the formation of neurovascular units. α1β1 integrins contact the endothelium with its extracellular matrix; similarly, α6β4 integrins stabilize the contact of astrocyte projections with the laminin-5 of the microvascular matrix. β2 integrins mainly mediate the adhesive interaction of leukocytes, while β3 integrins (cytoadhesins), including platelet glycoprotein αIIbβ3 (glycoprotein IIb-IIIa), are involved in the formation and stabilization of a blood clot. In response to brain ischemia, α1β1 and α6β4 integrins disappear very quickly in the ischemic region, which is explained by a decrease in their biosynthesis. Neurovascular units in this situation disintegrate (101, 102). According to the main structural pattern, integrins found in leukocytes consist mainly of those with the α and β2 subunits. The β2 subunit (also referred to as CD18) is homogenous, but there are a number of types of α subunits, e.g. 11a, 11b, and 11c. Thus, we can distinguish diverse types of CD18 integrins on the basis of their α-subunit. CD18/CD11a integrin is expressed in all leukocytes, and CD18/CD11b is expressed in neutrophils, monocytes and NK cells (100, 103, 104). These are the two leukocyte integrins most studied for their role in cerebral ischemia/reperfusion. Leukocyte activation increases the affinity of integrins for specific ligands on the surface of activated endothelia. Factors activating the expression of leukocyte integrins are IL-8 and MCP-1. The binding of an integrin to a ligand causes a conformational change in its intracellular domain, which interacts with elements of the cytoskeleton, giving the cell the opportunity for tight adhesion and migration through the vessel wall (100). In experimental studies on animals, it has been shown that the administration of antibodies against CD11a and CD18 reduced the extent of post-stroke damage and reduced the number of infiltrating neutrophils (105). It has also been shown that the expression of CD11a and CD18 in leukocytes was significantly increased in patients with stroke and transient ischemic attack. On the other hand, some studies have shown no increase in CD11b expression in stroke patients, and that the use of integrin-targeting therapy (anti-CD18/CD11b antibodies) in patients was not effective (103, 106, 107). Similar results were also obtained in studies concerning alpha-4 integrins (α4), which are crucial for the transvascular egress of lymphocytes T. Initially, using antibody blockade of α4, animal experiments showed promising outcomes, including a significant reduction in the size of the brain post-ischemic damage in normotensive and hypertensive rats (108). However, subsequent experimental studies yielded negative results (109). Finally, clinical trials (ACTION and ACTION II) with the use of monoclonal antibodies targeting α4 within the VLA-4 molecule (natalizumab) showed no therapeutic effect in patients with ischemic stroke (110, 111).

Immunoglobulin superfamily (IgSF) molecules mediate tight interactions between leukocytes and endothelia. Among them, the role of ICAM-1 has been the relatively most researched in the pathophysiology of brain ischemia/reperfusion. ICAM-1 is a molecule constitutively expressed in the endothelium, but its expression significantly increases during stroke in response to ischemic conditions and the stimulation of the endothelium with cytokines (IL-1, TNF-α, gamma interferon), reaching peak concentrations 12–24 hours after reperfusion (112, 113). Clinical tests showed an increase in ICAM-1 concentration in the blood serum and cerebrospinal fluid of patients after stroke (114). Experiments in mice in which the ICAM-1 gene has been knocked out showed an improvement in cerebral microcirculation, a decrease in the volume of post-stroke necrotic tissue, and a decrease in the number of infiltrating leukocytes after focal cerebral stroke with reperfusion compared to mice in the control group. The blockade of ICAM-1 with specific antibodies also reduced infarction and leukocyte infiltration of brain tissues in rats and rabbits following experimental stroke (112, 115, 116). Specific antigens for ICAM-1, such as LFA-1 and Mac-1, are found on the surface of leukocytes (117). In brain ischemia/reperfusion experiments on mice with a *null* mutation in the LFA-1 and Mac-1 genes, no effect on the adhesion process of leukocytes 4 hours after reperfusion was shown, while such an effect appeared after 24 hours, which correlated with the time of ICAM-1 peak density at the cell membranes of endothelial cells (92, 118). Despite promising results from preclinical studies, clinical trials using antibodies against ICAM-1 (Enlimomab) and Mac-1 (LeukArrest) were unsuccessful (119, 120). The roles of other molecules in the immunoglobulin superfamily in ischemic stroke are very poorly understood, and experimental studies have produced inconsistent results. In patients with stroke, the concentration of VCAM-1 in blood and its expression in endothelial cells and astrocytes in the ischemic region of the brain were significantly increased (121). However, experiments in rats and mice have shown that blocking VCAM-1 molecules with specific antibodies did not reduce the magnitude of post-stroke damage (122). Likewise, the role of ICAM-2 in brain ischemia/reperfusion is unknown. This molecule is expressed in activated endothelia and in nonactivated and activated platelets. It is speculated that ICAM-2 may be involved in the adhesion of platelets with leukocytes and the formation of PLAs during reperfusion (123). The last considered IgSF molecule – PECAM-1 is expressed in endothelial cells and most leukocytes. It can be reasonably postulated, that this molecule may be involved in the processes of adhesion and the transmigration of leukocytes through the endothelium during a ischemic stroke, as has been observed that the concentration of PECAM-1 increased significantly in the blood serum and cerebrospinal fluid of patients 24 hours after stroke insult (124). However, to date, there are few data on the role of PECAM-1 in experimental ischemia/reperfusion models. In one experimental study, PECAM-1 was found to control the transendothelial migration of neutrophils in a experimental mouse model of ischemic stroke, and antibody blockade of PECAM-1 during reperfusion ameliorated stroke severity in these mice (125).

### 2.3 Stroke Comorbidities – Their Associated Inflammation and Neurovascular Dysfunction

#### 2.3.1 Arterial Hypertension

Hypertension, defined as chronically elevated arterial blood pressure, leads to numerous changes in the blood vessels of the whole organism. Adaptive remodeling of the vascular walls or their hypertrophy or stiffness, a reduction of vessel caliber and changes in functional physiology, such as increased vascular resistance and circulatory perturbations, occur (126). The brain is an organ particularly affected by circulatory changes caused by hypertension. Several key mechanisms regulating cerebral blood microflow and maintaining brain energy homeostasis are disturbed, and therefore, the functions of NVUs are affected (127).

The dysfunction of the endothelium in the course of hypertension may be of various origins. One of the pro-dysfunctional pathways may be the production of oxygen free radicals by vascular systems: NOX (especially the NOX2 isoform), XO and the mitochondrial respiratory chain. It has been shown that the activity of NOX2 and XO is increased in hypertensive vessels (128). ROS react with NO to form peroxynitrite, thereby reducing the bioavailability of NO and causing an imbalance between this important vasodilator and the vasoconstrictors; disturbing the regulation of vascular wall tone and causing significant circulatory/energy deficits; and damaging proteins, lipids and nucleic acids (129). Systemic inflammation and neuroinflammation also play an important role in the pathophysiology of hypertension. Ongoing processes of ROS production and neuroinflammation likely damage various cells, which therefore release DAMPs signals (130). These molecules are well-known activators of TLR4. TLR4 receptors were the first TLRs proposed in the etiology of vascular inflammatory damage in hypertension (131). The activation of endothelial TLR4 leads, through the MyD88 protein, to the activation of the transcription factors AP-1 and NF-κB, which further enhance ongoing inflammation (132).

Although, it is difficult to state whether inflammatory processes are one of the primary or secondary causes of hypertension, it can now be assumed that inflammatory processes result from both primary and secondary causes of hypertension. The inhibition of NF-κB counters the increase in blood pressure that normally occurs in spontaneously hypertensive rats (SHRs), and decline high blood pressure in a neurogenic model of hypertension induced by deoxycorticosterone acetate administration in rats fed a high-sodium diet (133, 134). It is known that hypertension-linked inflammation most often occurs at a low-grade level, notwithstanding causing a number of unfavorable changes in the brain.

The inflammation marker considered to be significantly correlated with hypertension is CRP, and it has been confirmed that prehypertensive and hypertensive patients have elevated levels of CRP in blood serum (135). CRP molecule can stimulate circulating monocytes to express IL-6, IL-13 (interleukin 13) and TNF-α, and the expression of ICAM-1 and VCAM-1 adhesion molecules in the endothelium, including *via* activated NF-κB and AP-1. It has been shown that CRP can directly stimulate endothelial and smooth muscle cells to increase activation of NF-κB and AP-1 (136, 137). Moreover, elevated levels of chemokines, e.g., RANTES, were found in the serum of hypertensive patients (138, 139). The expression of some other adhesion molecules, e.g., P-selectin in platelets and endothelium and its antigen, PSGL-1 in the endothelium, may also take place through the action of the pro*-*hypertensive angiotensin II on the AT_1_R receptor (angiotensin II type 1 receptor) (140). Also immune cells residing in the brain – microglia/macrophages are sensitive to ongoing hypertension. As a result of the action of hypertension on microvessels in the brain, microglia are activated, expressing IL-1β and other pro-inflammatory molecules, such as IL-6 and TNF-α (141). Additionally, the specific class of macrophages residing in the vicinity of arterioles and venules, called perivascular macrophages (PVMs), plays a very important role in NVU dysfunction. Many studies on this issue have shown that the deletion of AT_1_R in PVMs partially attenuates BBB dysfunction, which results from NOX2 activity elevation. In contrast, the downregulation of AT_1_R in cerebral endothelial cells completely prevented BBB disruption. The results indicate that while endothelial AT_1_R, mainly in arterioles and venules, initiates BBB disruption in hypertension, PVMs are required for the full expression of the dysfunction (142). Another important molecule involved in hypertension and BBB damage is the CD36 receptor. In stroke-prone SHRs, increased expression of CD36 in microglia and associated BBB lesions were demonstrated, indicating a pro-inflammatory role of CD36 in the brain under hypertensive conditions (143). Importantly, CD36 receptor may be involved in the activation of astrocytes following brain ischemia, contributing significantly to the progression of inflammatory phenomena after a stroke onset (144).

The pro-inflammatory environment, formed in the brain as a result of the above processes (i.e., the accumulation of ROS, DAMPs, pro-inflammatory cytokines and chemokines; BBB weakening; and the activation of resident and systemic immune cells), promotes the formation of subsequent changes in brain tissues. Under the described conditions, the transmigration of systemic leukocytes may be promoted by an overestimated expression of PECAM-1, which in turn distinctly intensifies the weakening of the BBB (145). In hypertensive patients and in animal hypertension models, elevated concentrations of MMP-9 were also found, which indicates ongoing processes of leukocyte infiltration into the tissues (146). Inflammatory processes are also often associated with vasogenic edema. However, during low-grade neuroinflammation, the activation of astrocytes, astrocytic AQP4 overexpression and the development of mild cytotoxic edema should be taken into account – as has been confirmed that astrocytes in SHRs have been shown to increased expression of GFAP (147). Moreover, overexpresion of AQP4 in SHR brains was also detected (148).

All the above changes indicate a significant activation of astrocytes, a tendency toward cytotoxic edema, possible pressure on microvessels and disturbed communication with neurons. In this situation, the functions of NVUs (i.e., neurovascular coupling, NVC) will undoubtedly be disturbed, and there is numerous experimental evidence confirming this assumption; for a detailed review, see (149). Several experimental studies also indicate a significant increase in post-ischemic brain damage in hypertensive animals (150–153).

#### 2.3.2 Diabetes Mellitus

Diabetes mellitus is a chronic metabolic disease caused by insufficient insulin production (type 1 diabetes mellitus, T1DM) and/or a decrease in the tissue response to available insulin (type 2 diabetes mellitus, T2DM). This results in an elevation of blood glucose concentration, and in T2DM, also of insulin. The role of insulin resistance in the course of T2DM and the resulting hyperglycemia and hyperinsulinemia have been studied intensively in terms of causing dysfunction and damage to blood vessels at the level of the endothelium. Diabetic vascular complications affect many organs, including the brain-cerebral circulatory system and also the cerebral parenchyma (154). The brain is an organ that is significantly sensitive to inadequate blood glucose levels and to circulatory disturbances (155). A remodeling, stiffening of the vascular walls, and reduction of vessel caliber with changes in cerebral perfusion occur during diabetes (156).

The etiology of these diabetic complications is complex, alike as it is in the case of arterial hypertension and hyperlipidemia. Possible pathways of endothelial damage may involve ROS production and inflammatory processes. The production of ROS can take place in blood vessels through the activity of NOX, XO and the mitochondrial respiratory chain (157, 158). The decrease in NO bioavailability due to the generation of ROS/peroxynitrite and damage to biomolecules are important consequences leading to endothelial dysfunction. Oxidative stress and low-grade inflammation are recognized as important factors in the progression of T2DM and its vascular complications. Affected endothelial cells can release DAMP molecules, activating TLR4 and further potentiating inflammation (159). The specific DAMP signal – the peculiar effect of hyperglycemia is the formation of AGEs (advanced glycation end products), including proteins or lipids that, when exposed to an elevated glucose concentration, become covalently glycated. AGEs can stimulate RAGE, CD36 and TLR4 receptors, the pathways of which significantly contribute to the progression of inflammation in diabetes, particularly through NF-κB activity (160, 161). Additionally, the activation of MMP-9 in the brain and osmotic disturbances resulting from abnormal blood glucose levels weaken the BBB (162).

Inflammatory processes during diabetes are manifested by an increase in the CRP concentration in blood serum, the production of pro-inflammatory molecules, and the activation of immune system cells. CRP exerts a direct pro-inflammatory effect on endothelial and smooth muscle cells, inducing NF-κB and the expression of adhesion molecules (136, 137, 163). During hyperglycemia, NF-κB is significantly active in both types of tissues, resulting in the transcription of pro-inflammatory cytokines and an increase in leukocyte adhesion (164, 165). The expression of adhesion molecules in the endothelium in animal models of diabetes and in diabetic patients was found to be enhanced compared to nondiabetic control groups. Additionally, pro-inflammatory proteins such as IL-6 and MCP-1 show higher serum levels in diabetic patients (166, 167). It has been demonstrated in excellent study targeting neuroinflammation and BBB disorders (using T1DM and T2DM murine models) that diabetic hyperglycemia enhances BBB permeability and memory loss (Y maze and water maze tests) and elevated expression of inflammatory molecules in the brain, a.o.: MMP-9, VCAM-1, E-selectin, TNF-α, and CXCL2. Activation of microglia has also been shown (168). Brain microglia play an important role in diabetic cerebral injury. In murine models of diabetes mellitus, the activation and proliferation of microglia were observed in the brain, and activated microglia largely contributed to neuroinflammation and oxidative stress (169).

As in the case of hypertension, diabetes also causes the activation of cerebral astrocytes, which is manifested by the increased expression of GFAP by these cells. However, unlike in hypertension and hyperlipidemia, astrocytic AQP4 levels are lower in diabetic animals than in normoglycemic ones (170). Both of these results indicate the possibility of communication disorders between astrocytes, blood vessels and neurons, which, combined with the abovementioned processes of ROS and DAMPs production, endothelial damage, the release of inflammatory molecules, microglial activation, indicates the creation of an environment that strongly promotes NVUs dysfunction in the brain. NVC abnormalities in the brain have been detected in both an animal models of diabetes and in diabetic patients (171). The initial diabetic NVUs dysfunctions, may be a significant cause of a worsen stroke outcomes, which has been shown in several studies in experimental models of cerebral ischemia (150, 172–174).

#### 2.3.3 Hyperlipidemia

Hyperlipidemia is most commonly defined as elevated plasma cholesterol levels, which may be associated with elevated plasma triglycerides. Lipids are transported by the plasma in the form of lipoproteins (e.g., LDL – low-density lipoprotein); hence, hyperlipidemia is most commonly diagnosed in patients based on elevated levels of plasma lipoproteins. Both hypercholesterolemia and hypertriglyceridemia can cause the dysfunction of the whole body vasculature, including cerebral blood vessels (10, 175).

In experimental research, one of the frequently used animal models of hyperlipidemia is the apolipoprotein E-deficient murine model (Apoe^-/-^, apolipoprotein E is one of the protein components of lipoproteins such as LDL and is involved in lipid transport to tissues). In interesting studies on these mice fed a high-fat diet (HFD), elevated plasma lipid levels disturbed the regulation of cerebral blood flow. Cerebral microcirculation responses to hypercapnia and functional stimulation (NVC) were impaired, while no atherosclerotic plaques were found in these animals (176). In other experiments in the same mouse model, a decrease in the microcirculatory response to acetylcholine was shown, which was improved with the use of a free radical scavenger or NOX inhibitor (177). In another hyperlipidemia model, after 10 weeks of HFD feeding in mice, both cerebral macrovessels and microvessels underwent remodeling, including an increased cerebrovascular tortuosity index and decreased arterial inner diameters. This remodeling could be mediated, at least in part, by MMP-9, as an HFD induced similar levels of hyperlipidemia in MMP-9-deficient mice, but there was no cerebrovascular remodeling (178). Nevertheless, the exact mechanistic insights into how elevated plasma lipids damage cerebral blood vessels and impair neurovascular coupling are not fully clear.

Some studies have shown an increased level of CRP in the blood plasma of patients with hypercholesterolemia (179). This result may indicate that these individuals have low-grade background inflammation, similar to hypertension and diabetes. Likewise, oxidative processes can also occur. There is evidence of oxidative modification of LDL mediated by lipoxygenase (expressed by macrophages) and/or myeloperoxidase (expressed by neutrophils and monocytes), which generate the product of LDL oxidation – oxidized LDL molecule (oxLDL) (180). It should be emphasized that some authors assign oxLDL to the category of DAMP molecules, there is also evidence pointing to a pro-HMGBl action of oxLDL in cerebral vessels, which indicate on the generation of an alarm signal to excite the effectors of the immune system (181). OxLDL induces the assembly of the TLR 4/6 heterodimer, involves CD36, and consequently induces the NF-κB and AP-1 pathways and the secretion of pro-inflammatory molecules, e.g., cytokines and chemokines (182).

Low-grade inflammation in the course of hyperlipidemia is a likely cause of NVUs dysfunction in the brain. Excellent experimental studies in wild-type C57/BL6J mice fed a high-cholesterol diet (HCD) showed a tremendous increase in rolling and adherent leukocytes and in platelets in the cerebral venules. P-selectin immunoneutralization attenuated the interactions of leukocytes and platelets with endothelia. The same study also showed that in mice in which one of the subunits of NOX had been knocked out and fed an HCD diet, the number of rolling and adherent leukocytes in the cerebral venules decreased significantly compared to that in wild-type mice fed an HCD diet (14). Moreover, it has been shown that oxLDL can induce the expression of ICAM-1 and VCAM-1 adhesion molecules in the human endothelium (183). Studies with HCD-fed Apoe^-/-^ and wild-type control mice showed a significant increase in IL-6 expression in the hippocampus and cerebral cortex in both strains (184). In studies carried out on rats fed hypercholesterolemic diets, an increase in the concentration of IL-1 and TNF-a in blood plasma was detected after the first week, and an increase in GFAP expression in the brain, while after the third week, a decrease in expression of claudin-5 in the brain was detected (185). In other studies carried out on rats fed an HFD, an increase in GFAP expression as well as AQP4 and microglial activation were detected (186). However, in studies in wild-type mice fed an HCD diet, no significant changes in GFAP expression in the brain were detected after three months. The same result was obtained in transgenic mice overexpressing transforming growth factor-ß1; however, in these mice, the HCD diet caused spatial learning deficits and increased the number of thinned blood vessels in the internal capsule. The effects of the HCD on spatial learning and the studied vascular morphology changes in these mice were reduced when simvastatin, a drug that lowers blood cholesterol, was administered during the diet (187). In other studies, HCD-fed C57/BL6J wild-type mice showed an increase in GFAP expression in the hippocampus and the cerebral cortex and AQP4 in the hippocampus. There was no microglial activation (188). All of the above studies were conducted on males, interesting results of the influence of an HFD on inflammatory markers in the brains of wild-type C57BL/6J female mice (10–12 weeks old) were provided by the study of Peterson TC., et al., which showed that 6 weeks of the HFD did not induce changes in GFAP expression or microglial activation in the cerebral cortex (189). The above differences in the results of studies on the impact of an HFD on GFAP expression and microglia status may result from differences between the experimental procedures and animal models used. In the case of the study by Peterson TC., et al., one of the results was that the HFD had no effect on GFAP expression, possibly due to the protective effect of female sex hormones counteracting low-grade neuroinflammation. Estrogens have both neuroprotective and anti-inflammatory effects, e.g., 17β-estradiol has been shown to have an inhibitory effect on GFAP expression in mice after traumatic brain injury (190). Nevertheless, extensive studies in rats and mice fed an HFD and observations of obese patients found that rodents had increased GFAP expression in the hypothalamus and obese patients had features of gliosis in the hypothalamus (191).

In summary, all the above study results strongly suggest that processes that damage NVUs in the brain take place in the course of hyperlipidemia. This initial disturbance in NVUs may impart an important contribution to the pathophysiology of ischemic stroke, as hyperlipidemia has been shown to increase post-ischemic brain damage in experimental animal models of cerebral ischemia (174, 192–194).

## 3 Summary: Cerebral Ischemia/Reperfusion Cascade and Comorbidities – The Integrated Perspective

The insufficient blood supply to the brain initiates the ischemic cascade. In a focal stroke, tissues located in the ischemic core are the first to be destroyed, as neurons located in this area undergo necrosis within a few minutes due to the disruption to energy and ion homeostasis, and the neurovascular units in this zone decay. At the same time, harmful factors such as glutamic acid and potassium ions, released in excessive concentrations from the infarct core, reach the surrounding penumbral area and cause gradual recruitment of the penumbra to the infarct core through excitotoxicity processes and recurrent peri-infarct depolarizations, which cause the production of ROS and RNS, increased expression MMP-9 and MMP-2, disturbances in communication between components of the neurovascular units, and further hemodynamic disturbances during reperfusion. Dead/dying cells release DAMPs signals, leading to the mobilization of the innate immune system through the activation of pattern recognition receptors (TLR4, RAGE, and CD36) in microglia, astrocytes, endothelia, and peripheral leukocytes recruited to the infarction. Intracranial edema, resulting from changes in AQP4 expression (upregulation and/or downregulation) and BBB weakening, strongly contributes to the progression of cerebral damage and inflammation.

**Figure 1:**
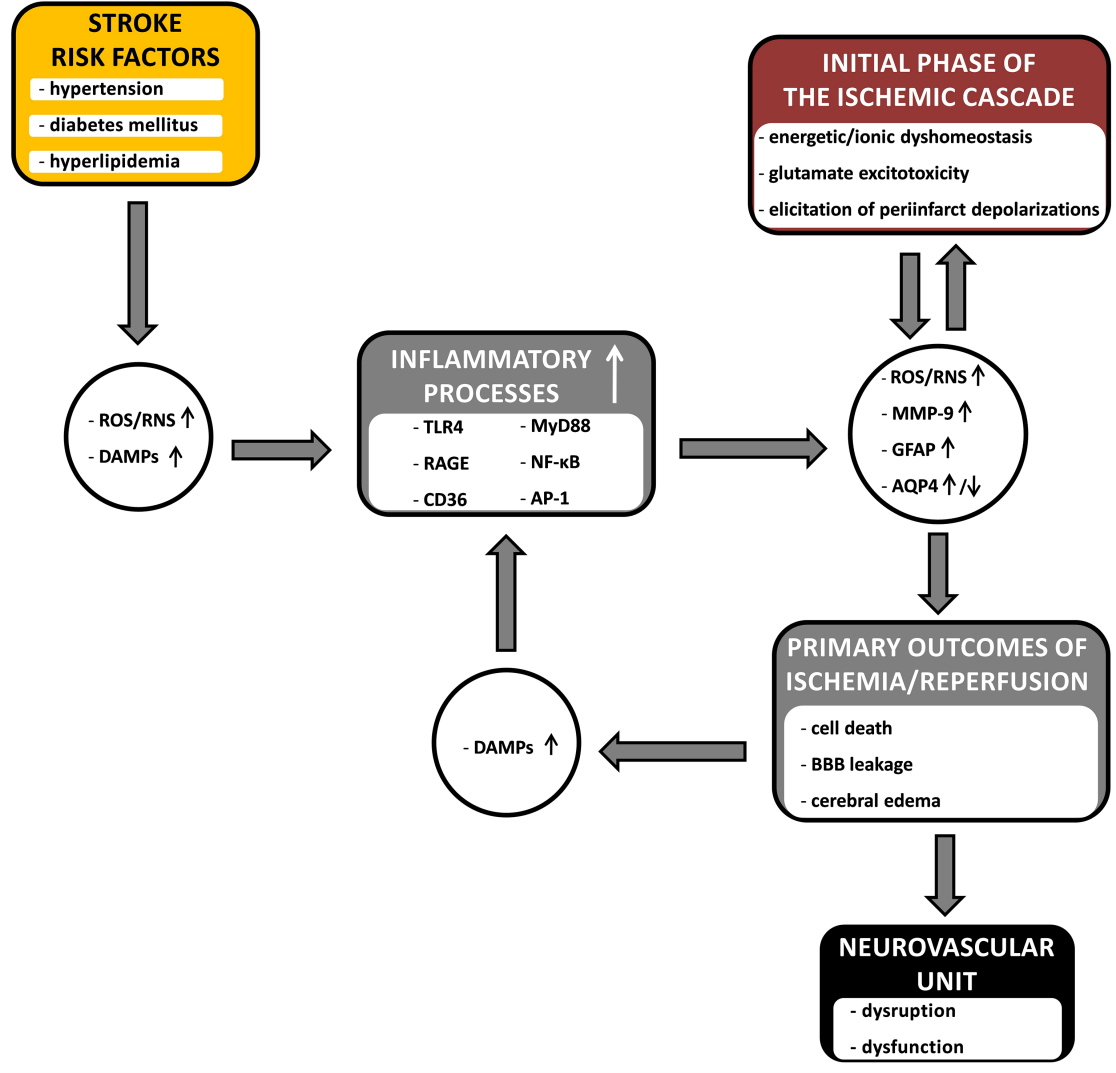
The diagram showing the hypothetical links between common stroke comorbidities (arterial hypertension, diabetes mellitus and hyperlipidemia), inflammatory processes and the course of cerebral ischemia/reperfusion cascade. The comorbid risk factors enhance the influence of the inflammatory processes on the course of the ischemia/reperfusion cascade, contributing to the processes leading to the damage and dysfunction of the neurovascular unit in the penumbra. Inflammatory processes, together with the primary outcomes of the ischemic cascade, form a vicious cycle – where they constitute an amplifying point, and the connecting bridge point, between risk factors and the ischemic cascade. The detailed description is provided in the text. ROS, reactive oxygen species; RNS, reactive nitrogen species; DAMPs, danger associated molecular patterns; TLR4, toll like receptor 4; RAGE, receptor for advanced glycation end products; CD36, platelet glycoprotein 4; MyD88, myeloid differentiation primary response protein (innate immune signal transduction adaptor); NF-κB, nuclear factor kappa B; AP-1, activator protein 1; MMP-9, matrix metalloproteinase-9; GFAP, glial fibrillary acidic protein; AQP4, aquaporin-4; BBB, blood-brain barrier; ↑, upregulation; ↓, downregulation.

Concomitant stroke risk factors (as dealt with here: arterial hypertension, diabetes mellitus and hyperlipidemia) strongly drive these inflammatory processes; because these factors are often present for a long time before a stroke, causing low-grade background inflammation throughout the body and, as we have seen, in the brain, negatively affecting the functioning of neurovascular units. **Figure 1** summarizes proposed pathways along which the risk factors for stroke, clinically coexisting severally or in clusters, contribute to the course of the cerebral ischemic/reperfusion cascade, enhancing its progression and destructive effects on the neurovascular units in the ischemic penumbra. The key bridging factors proposed here are inflammatory processes. Low-grade inflammation in the brain before a stroke insult occurs through the activation of mostly the same inflammatory pathways that are activated by the ischemia/reperfusion cascade, i.e., the production of ROS/RNS, DAMPs and the activation of TLR4, RAGE and CD36 receptors, leading to an increase in the expression of MMP-9 and GFAP and changes in AQP4 expression, which are important markers of ongoing NVU-damaging processes. Hence, presensitized neurovascular units are more easily disintegrated and become dysfunctional in the face of damaging factors in the ischemic/reperfusion cascade in the penumbra. Initial sensitization of NVUs as well as driving and strengthening the processes of the ischemia/reperfusion cascade are very likely causes of the great difficulties in the treatment of acute stroke patients. What is equally important is that these factors are also likely the cause of the failure of attempts to introduce neuroprotectants into clinical practice, as most of the *in vivo* studies were conducted on healthy and young animals that did not show initial sensitization of NVUs to the ischemia/reperfusion cascade.

As a conclusion, targets for immunomodulatory stroke treatment that can be proposed in the context of the issues reviewed include the pattern recognition receptors that recognize DAMPs. This is because, as was suggested, they may constitute the main bridge between the common stroke risk factors and the ischemic cascade in the brain, and its activation is one of the first stages of neuroinflammatory processes in the course of a stroke. Therefore, reduction of their activation may be crucial for the therapy. Such management may also prove to be safer than typical immunosuppression strictly targeted cellular response, due to the fact that it may increase potential infections, e.g. in the lungs. Arguably, an effective treatment of a ischemic stroke should consist of three elements progressing in succession: neuroprotection and immunomodulation applied as soon as possible before recanalization of the plugged vessel, effective recanalization, and then neuroprotective and immunomodulating treatment during reperfusion (195). Neuroprotection and immunomodulation are both important considering the classic concept of the hemodynamic penumbra and the concept of the “inflammatory penumbra” (196, 197). The inflammatory penumbra spreads beyond the hemodynamic penumbra, increasing the probability of more brain damage by acting through inflammation in the brain.

In the context of the above-mentioned conclusions, there is also a need for preclinical research using animal models that are more relevant to the general health background-condition of patients. Animal models should emphasize the coexistence of several risk factors concerning a single patient, as they often occur jointly (e.g. hypertension-diabetes-obesity), even if mild, and on the duration of their impact on the body. Furthermore, theoretical considerations when designing a therapy should also take into account this type of background and should be directed at several components of the whole neurovascular unit (195). Presumably, the treatment effectiveness confirmed on such experimental models gives a greater chance of its success in the clinic.

## Author Contributions

ŁP wrote the manuscript and approved it for publication.

## Funding

This article was financially supported from the Mossakowski Medical Research Institute, Polish Academy of Sciences funds.

## Conflict of Interest

The author declares that the research was conducted in the absence of any commercial or financial relationships that could be construed as a potential conflict of interest.

## Publisher’s Note

All claims expressed in this article are solely those of the authors and do not necessarily represent those of their affiliated organizations, or those of the publisher, the editors and the reviewers. Any product that may be evaluated in this article, or claim that may be made by its manufacturer, is not guaranteed or endorsed by the publisher.
